# Chemical Composition, Allelopathic, Antioxidant, and Anti-Inflammatory Activities of Sesquiterpenes Rich Essential Oil of *Cleome amblyocarpa* Barratte & Murb.

**DOI:** 10.3390/plants10071294

**Published:** 2021-06-25

**Authors:** Ahmed M. Abd-ElGawad, Abdelbaset M. Elgamal, Yasser A. EI-Amier, Tarik A. Mohamed, Abd El-Nasser G. El Gendy, Abdelsamed I. Elshamy

**Affiliations:** 1 Department of Botany, Faculty of Science, Mansoura University, Mansoura 35516, Egypt; yasran@mans.edu.eg; 2 Plant Production Department, College of Food & Agriculture Sciences, King Saud University, P.O. Box 2460, Riyadh 11451, Saudi Arabia; 3 Department of Chemistry of Microbial and Natural Products, National Research Centre, 33 El-Bohouth St., Dokki, Giza 12622, Egypt; algamalnrc_2010@nrc.sci.eg; 4 Chemistry of Medicinal Plants Department, National Research Centre, 33 El-Bohouth St., Dokki, Giza 12622, Egypt; ta.mourad@nrc.sci.eg; 5 Medicinal and Aromatic Plants Research Department, National Research Centre, 33 El Bohouth St., Dokki, Giza 12622, Egypt; ag.el-gendy@nrc.sci.eg; 6 Chemistry of Natural Compounds Department, National Research Centre, 33 El Bohouth St., Dokki, Giza 12622, Egypt

**Keywords:** phytotoxicity, environmental factors, volatile oils, *Cleome* genus, anti-inflammation

## Abstract

The integration of green natural chemical resources in agricultural, industrial, and pharmaceutical applications allures researchers and scientistic worldwide. *Cleome amblyocarpa* has been reported as an important medicinal plant. However, its essential oil (EO) has not been well studied; therefore, the present study aimed to characterize the chemical composition of the *C. amblyocarpa*, collected from Egypt, and assess the allelopathic, antioxidant, and anti-inflammatory activities of its EO. The EO of *C. amblyocarpa* was extracted by hydrodistillation and characterized via gas chromatography–mass spectrometry (GC-MS). The chemometric analysis of the EO composition of the present studied ecospecies and the other reported ecospecies was studied. The allelopathic activity of the EO was evaluated against the weed *Dactyloctenium aegyptium*. Additionally, antioxidant and anti-inflammatory activities were determined. Forty-eight compounds, with a prespondence of sesquiterpenes, were recorded. The major compounds were caryophyllene oxide (36.01%), hexahydrofarnesyl acetone (7.92%), alloaromadendrene epoxide (6.17%), myrtenyl acetate (5.73%), isoshyobunone (4.52%), shyobunol (4.19%), and *trans*-caryophyllene (3.45%). The chemometric analysis revealed inconsistency in the EO composition among various studied ecospecies, where it could be ascribed to the environmental and climatic conditions. The EO showed substantial allelopathic inhibitory activity against the germination, seedling root, and shoot growth of *D. aegyptium*, with IC_50_ values of 54.78, 57.10, and 74.07 mg L^−1^. Additionally, the EO showed strong antioxidant potentiality based on the IC_50_ values of 4.52 mg mL^−1^ compared to 2.11 mg mL^−1^ of the ascorbic acid as standard. Moreover, this oil showed significant anti-inflammation via the suppression of lipoxygenase (LOX) and cyclooxygenases (COX1, and COX2), along with membrane stabilization. Further study is recommended for analysis of the activity of pure authentic materials of the major compounds either singularly or in combination, as well as for evaluation of their mechanism(s) and modes of action as antioxidants or allelochemicals.

## 1. Introduction

Wild plants are considered green factories for the synthesis of thousands of bioactive compounds that have various biological activities and are integrated into the treatment of various diseases, controlling weeds as biocides, as well as being used in agricultural, industrial, and pharmaceutical applications [1]. The use of natural bioactive compounds instead of synthetic chemicals fascinates scientists, researchers, and policymakers because they are renewable, degradable, safe, and low toxic [2,3].

Essential oils (EOs) are the main constituents of the members of the plant kingdom [4]. Historically, EOs represented one of the main resources of significant pharmaceutical and biological agents because of their complicated chemical composition, basically as isoprenoids [5]. Many biological potentialities for EOs have been described as hepatoprotective, anticancer [4], antioxidant [6,7], anti-inflammatory [8], anti-aging [4], antipyretic [6], and antimicrobial [9], in addition to allelopathy [10,11,12].

*Cleome amblyocarpa* Barr. & Murb. (Syns: *Cleome arabica* var. *amblyocarpa* (Barratte & Murb.) Ozenda, *Cleome africana* Botsch., or *Cleome daryoushiana* Parsa) is a herbaceous plant of the Cleomaceae family [13]. It is widely growing in sandy or stony habitats of desert along with North Africa [14]. This plant has been used in folk medicine for the treatment of various diseases such as diabetes and colic, as a stomachic therapy, rheumatic fever, scabies, and inflammation [15]. Various bioactive compounds have been isolated from *C. amblyocarpa*, such as flavonoids and glucosinolates [16], saponins [17], triterpenoids [18]. Therefore, this herb has been reported to possess various biological activities, including anti-inflammatory [16], anti-COVID-19 [17], genotoxicity [9], antidiabetic and antioxidant [19], and antimicrobial effects [20].

The EOs of several species of genus *Cleome* have been studied, such as *C. droserifolia* [21], *C. trinervia* [22], *C. monophylla* [23], and *C. serrata* [24], *C. coluteoides* [25]. However, few studies have dealt with the EO of *C. amblyocarpa* [26]. Additionally, and to the best of our knowledge, the allelopathic activity of the EO of *C. amblyocarpa* has not been studied before. Therefore, the present study aimed to (1) characterize the chemical composition of the EO isolated from the Egyptian ecospecies of *C. amblyocarpa*, and (2) evaluate the allelopathic activity, antioxidant, and anti-inflammatory activities of its EO.

## 2. Results and Discussion

### 2.1. EO Composition of C. amblyocarpa

**Figure 1:**
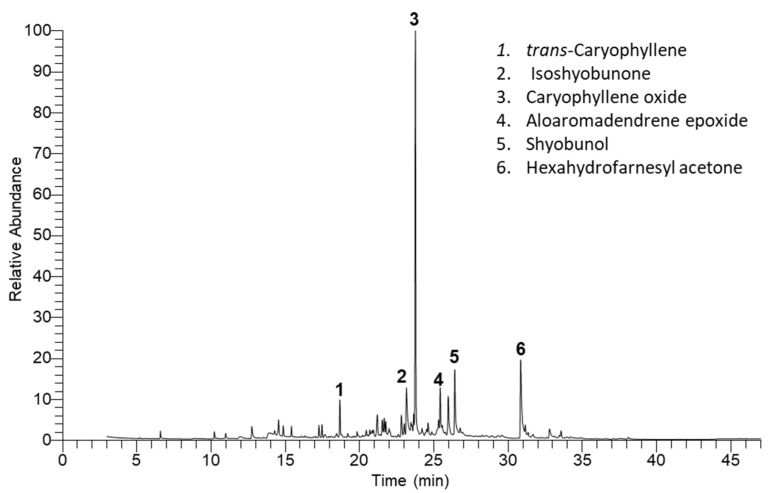
Chromatogram of the chemical compounds identified via GC-MS in the EO of *Cleome amblyocarpa* above-ground parts. The major compound peaks are numbered (1–6).

The hydrodistillation of the air-dried powder of the above-ground parts of *C. amblyocarpa* yielded 0.38% (*v/w*) of golden-yellow oil. Depending upon the GC-MS analysis (Figure 1), 48 compounds were characterized, representing 97.17% of the total mass (Table 1). The Egyptian ecospecies of the present study yielded higher EO yield compared to Iranian [27] and Saudi [28] ecospecies, which had 0.20 and 0.21%, respectively. This variation in oil production could be ascribed to environmental, climatic, or genetic factors [5,6,29,30,31]. The term “ecospecies” means that species of the plant can be divided into several ecotypes (a genetically distinct population of plants that is growing in a particular habitat).

**Figure 2:**
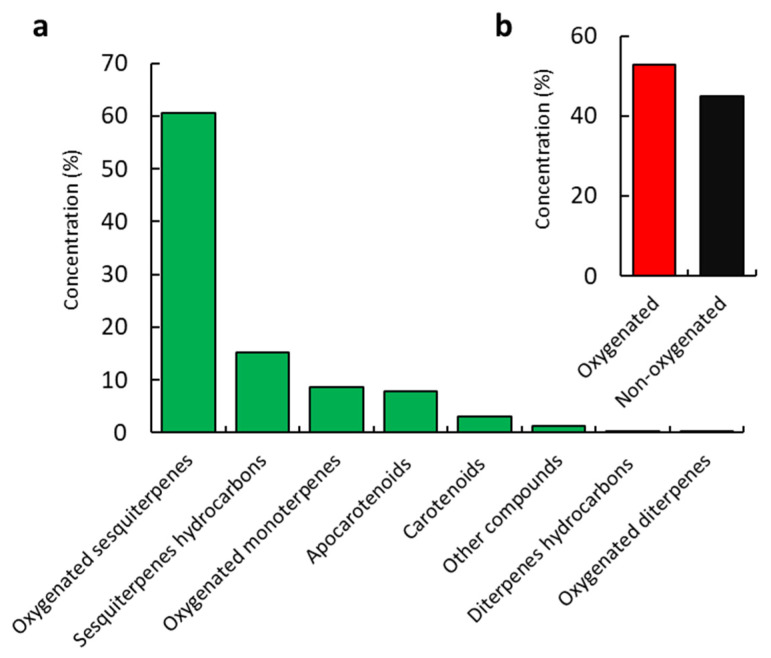
Concentrations of various identified classes of the chemical compounds of the *Cleome amblyocarpa* EO (**a**) and the percentage of oxygenated and non-oxygenated compounds (**b**).

In the EO of *C. amblyocarpa*, six classes of components were determined, comprising oxygenated sesquiterpenes, sesquiterpenes hydrocarbons, oxygenated monoterpenes, diterpenes hydrocarbons, oxygenated diterpenes, apocarotenoid-derived compounds, carotenoid-derived compounds, and other compounds (Figure 2). These compounds pooled as 81.80% oxygenated compounds and 15.37% as non-oxygenated compounds. From overall mass, terpenoids represented the main constituents with a relative 84.88% with a preponderance of sesquiterpenes (75.77%), a remarkable concentration of monoterpenes (8.72%), and traces of diterpenes (0.39%). The abundance of terpenoids in the EO of *C. amblyocarpa* was in agreement with the data reported for samples collected from Iran [27] and the United Arab Emirates [26]. In contrast, the plurality of sesquiterpenes was inconsistent with the Iranian *C. amblyocarpa*, in which diterpenoids were determined as the major class [27], and *C. amblyocarpa* collected from the United Arab Emirates, in which monoterpenes were the main constituents [26]. These significant variations of chemical composition might be ascribed to environmental circumstances (such as temperature, rainfall, soil factors, altitude, etc.) and genetic characteristics [11,30,32,33].

Sesquiterpenes were assigned as the main components involving the oxygenated compounds as majors (60.62%) in addition to a relative concentration of 15.15% of sesquiterpene hydrocarbons. Out of the 16 identified oxygenated sesquiterpenes, caryophyllene oxide (36.01%), alloaromadendrene epoxide (6.17%), isoshyobunone (4.52%), and shyobunol (4.19%) represented the major compounds, whereas *β*-cubebene (0.13%) was the minor compound. Caryophyllene oxide is a common major compound in several EOs derived from plants such as *Cullen plicata* [34], *Schinus polygamus* [35], *Curcuma sahuynhensis* [36]. Caryophyllene oxide was documented as a minor compound in the EO of Iranian *C. amblyocarpa* [27], and totally absent from the EO of *C. amblyocarpa* collected from the United Arab Emirates [26], Tunisia [37], and Saudi Arabia [28]. On the other hand, *trans*-caryophyllene (3.45%), and *α*-muurolene (2.30%) were found to be the main sesquiterpene hydrocarbons, whereas silphiperfol-5,7(14)-diene (0.13%) was determined as a minor component. *trans*-caryophyllene has been reported in trace amounts of the EO of the Iranian ecospecies of *C. amblyocarpa* [27], whereas it is completely absent from the EO of Saudi [28], Emirati [26], and Tunisian [37] ecospecies.

The oxygenated monoterpenes were represented by 8.72%, which contained seven compounds, with myrtenyl acetate (5.73%) and borneol (1.12%) as major compounds. These two compounds are totally absent from the other ecospecies of *C. amblyocarpa* [26,27,28,37]. On the other hand, low diterpene contents were determined and represented by two compounds, geranyl-*α*-terpinene (0.22%) and phytol (0.17%). However, diterpenes were absent from other ecospecies of *C. amblyocarpa*. In other species of *Cleome* genus, phytol was reported in a high concentration such as *C. monophylla* [23], *C. serrata* [24], and *C. serrata* [38].

Carotenoid-derived compounds were determined in a concentration of 3.03%, that represented only two compounds, dihydroedulan II (1.64%) and theaspirane A (1.39%). Only one apocarotenoid-derived compound, hexahydrofarnesyl acetone, was identified with a high relative concentration (7.92%), whereas it was completely absent from the other reported ecospecies of *C. amblyocarpa* [26,27,28,37]. Hexahydrofarnesyl acetone is a widely distributed major compound in the EOs of several plants such as *Bassia muricata* [10], *Heliotropium curassavicum* [33], *Hildegardia barteri* [39], *Trianthema portulacastrum* [40].

Finally, traces of other non-terpenoid components were characterized including only two compounds, *p*-isopropy-l-benzaldehyde (1.16%) and 9,12-octadecadienoic acid (0.18%).

### 2.2. Chemometric Analysis of the EOs from Different C. amblyocarpa Ecospecies

**Figure 3:**
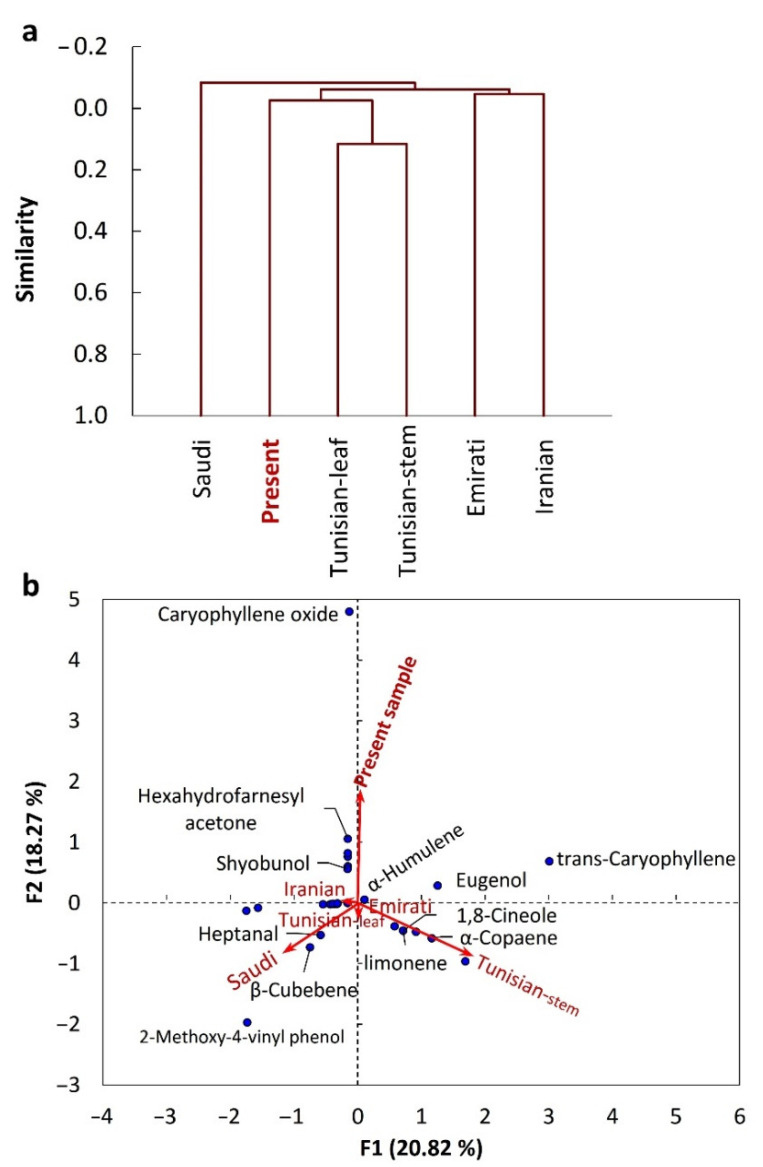
Chemometric analysis of various *Cleome amblyocarpa* ecospecies. (**a**) agglomerative hierarchical clustering (AHC), (**b**) and principal components analysis (PCA). (**F1**) and (**F2**) are factor 1 and 2.

The chemometric analysis of the EO composition of the present studied *C. amblyocarpa* and other reported ecospecies (Saudi, Iranian, Tunisian, and Emirati) was performed using cluster analysis and PCA (Figure 3). The cluster analysis revealed substantial variations among the studied ecospecies, and we can categorize them into three groups: group I comprising the present Egyptian and Tunisian ecospecies, group II containing Emirati and Iranian ecospecies, and finally the Saudi ecospecies separated alone as group III (Figure 3a). Interestingly, the chemometric analysis revealed that the EO compositions of ecospecies from the nearest countries were similar. This observation reflects the effect of environmental and climatic factors [30,32,33].

However, the present studied *C. amblyocarpa* ecospecies showed a correlation with the caryophyllene oxide, hexahydrofarnesyl acetone, and shyobunol, whereas the Tunisian ecospecies showed a correlation with ethyl 3-methylpentanoate, 7-epi-silphiperfol-5-ene, *α*-copaene, and 1,8-cineole (Figure 3b). The Saudi ecospecies was characterized by cis-dihydro carvone, 2-methoxy-4-vinyl phenol, and cubebene heptanal.

### 2.3. Allelopathic Effect of the C. amblyocarpa EO

**Figure 4:**
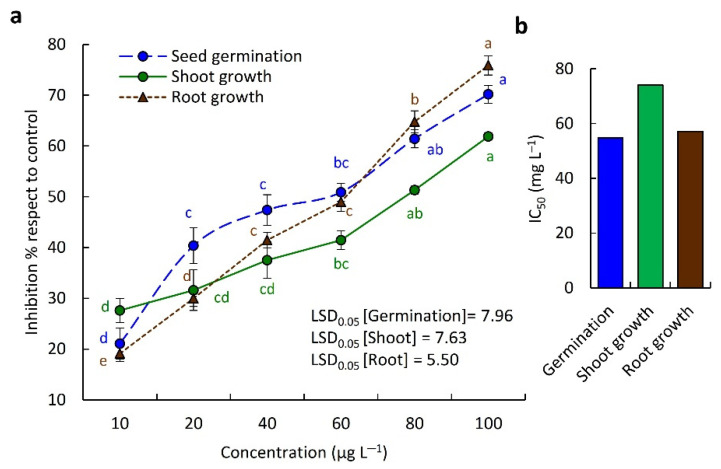
Allelopathic activity of the EO from the above-ground parts of *Cleome amblyocarpa* on the germination, root and shoot growth of *Dactyloctenium aegyptium*. (**a**) Various concentration and (**b**) IC_50_. Different letters within each line mean values significantly different at *p* < 0.05 (Tukey’s HSD test).

The EO of *C. amblyocarpa* showed significant allelopathic activity of the seed germination (*p* < 0.05) as well as the shoot and root development of *D. aegyptium* in a dose-dependent manner (Figure 4a). At the highest concentration (100 µg mL^−1^), the germination was inhibited by 70.18%, whereas the seedling root and shoot were reduced by 75.88% and 61.87%, respectively. Based on the IC_50_ values, the root was more affected than the shoot, where the roots had an IC_50_ value of 57.10 µg mL^−1^, and the root attained 74.07 µg mL^−1^ (Figure 4b). Root has been reported to be more affected by allelochemicals than the shoot. This observation was reported for many plant species such as *Deverra tortuosa* [41], *Teucrium polium* [42], *Calotropis procera* [5], *Ficus carica* [43], and *C. plicata* [34]. This could be ascribed to the direct contact of the root with the medium and the high permeability of root cells [34,44].

To the best of our knowledge, the allelopathic activity of the EO from *C. amblyocarpa* has not been studied yet. However, the aqueous, hexane, chloroform, and methanol extracts from *C. amblyocarpa* have been reported to inhibit lettuce germination and growth [45]. At a concentration of 6 g L^−1^, ethyl acetate showed a complete inhibition of lettuce growth, whereas *Peganum harmala*, *Raphanus sativus*, and *Silybum marianum* were more resistant. Additionally, Ladhari et al. [46] identified some terpenoids and flavonoids from *C. amblyocarpa*, where dammarane-type triterpenes showed strong allelopathic activity, and flavonoid compounds exhibited <50% inhibition of the targeted species.

In our results, the allelopathic activity of *C. amblyocarpa* EO could be attributed to the activity of a single or combination of the major identified compounds. Caryophyllene oxide is reportedly a major compound of the EOs with substantial allelopathic activity such as *C. plicata* [34], *H. curassavicum* [33], *Acroptilon repens* [47], *Teucrium arduini*, *T. montbretii* [48], and *Nepeta curviflora* [49]. On the other hand, the EO from *Launaea mucronata* and *L. nudicaulis* showed the presence of hexahydrofarnesyl acetone as a major compound, where it revealed significant allelopathic activity against the weed: *Portulaca oleracea* [30]. Additionally, the EO of *H. curassavicum* had hexahydrofarnesyl acetone as the main compound, and showed marked allelopathic activity on *Chenopodium murale* [33]. The major compound, alloaromadendrene epoxide, in the EO of the present *C. amblyocarpa* has also been reported as the main compound (7.32%) of the EOs from *Lactuca serriola* that showed allelopathic activity against the weed *Bidens pilosa* [50]; however, in the EO of *Calamintha nepeta*, it did not show significant allelopathic activity against *Raphanus sativus*, *Lepidium sativum*, *Sinapis arvensis*, *Triticum durum*, and *Phalaris canariensis* [51]. This inconsistency could be attributed to the resistance of the weeds and shows that allelochemicals are species-specific [52].

Generally, the oxygenated terpene compounds have been reported to possess allelopathic activity compared to the non-oxygenated compounds [6]. In the present study, the EO of *C. amblyocarpa* was very rich in oxygenated compounds (81.80%), which could explain the notable allelopathic activity.

### 2.4. Antioxidant Activity of C. amblyocarpa EO

**Figure 5:**
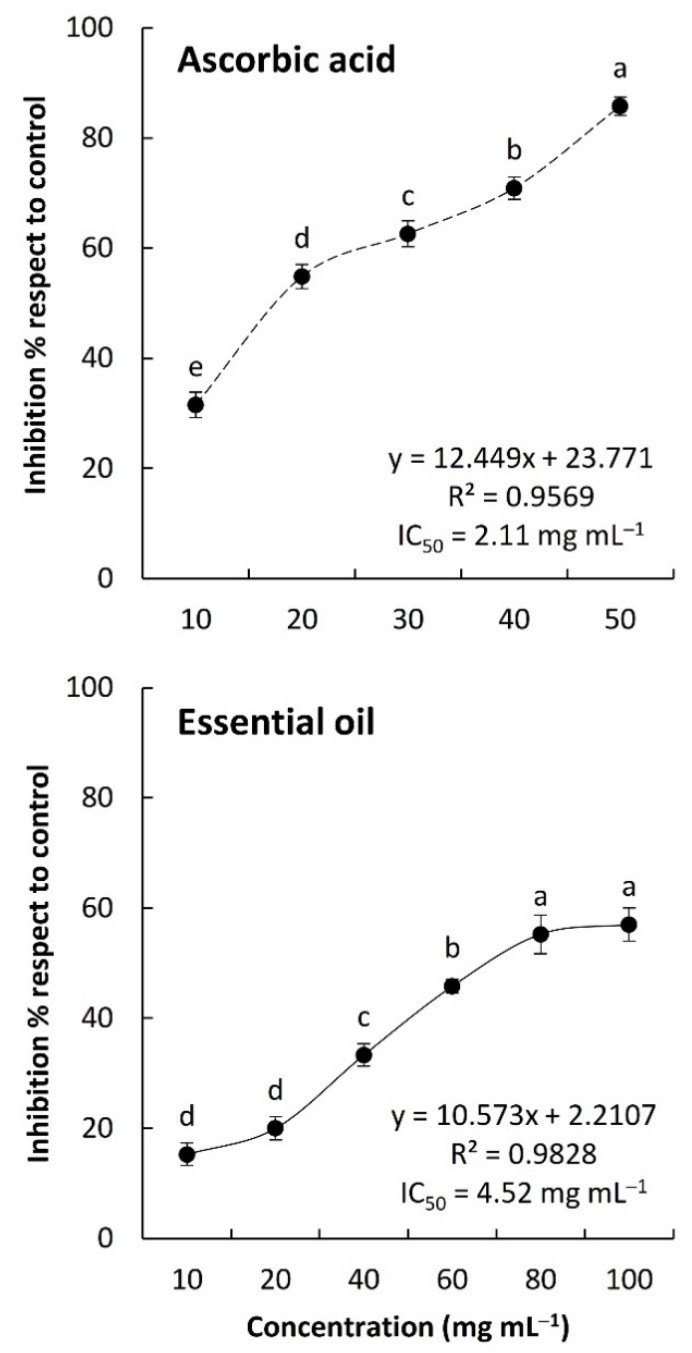
Antioxidant activity of the EO from the above-ground parts of *Cleome amblyocarpa*. Different letters of the line mean values significant at *p* < 0.05 (Tukey’s HSD test).

The activity of *C. amblyocarpa* EO in the reduction in the DPPH revealed significant antioxidant activity in a dose-dependent manner (Figure 5). At the lowest concentration of the EO (10 mg mL^−1^), the EO showed a 15.28% scavenging activity of the DPPH, whereas at the highest concentration (100 mg mL^−1^), the antioxidant activity was reduced by 3.7-fold of the lowest concentration of the EO. Based on the IC_50_, the *C. amblyocarpa* EO had a value of 4.52 mg mL^−1^ compared to 2.11 mg mL^−1^ of the ascorbic acid as a standard antioxidant. The antioxidant activity of EOs in the present study were lower than those reported from Tunisian ecospecies [37]. This could be ascribed to the variation in the EO chemical compositions (Table 1).

The free radical scavenging activities of plant extracts and/or EOs were directly correlated with the concentration of the oxygenation of their constituents due to the increase in the free electrons [53]. The present data revealed 81.08% of oxygenated compounds, which means that a wealth of free electrons can act to diminish free radicals in the evaluation reaction. More specifically, the activity of this EO may be attributed to the activity of major compounds, either singularly or in synergy. Caryophyllene oxide has been reported to possess antioxidant activity [34]. EOs rich in hexahydrofarnesyl, such as *B. muricata* [10] and *H. curassavicum* [33], showed substantial antioxidant activity. The EO extracted from *L. serriola* has been reported to be rich in alloaromadendrene epoxide and isoshyobunone, where it expressed strong antioxidant activity [50].

### 2.5. Anti-Inflammatory effect of C. amblyocarpa EO

**Figure 6:**
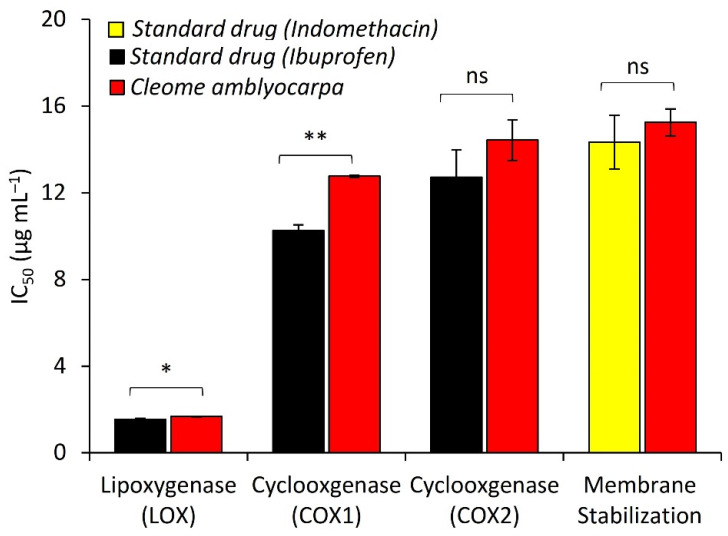
The anti-inflammatory activity of the EO of *Cleome amblyocarpa*, based on membrane stabilizing, and the inhibition of lipoxygenase (LOX) and cyclooxygenase (COX1 and COX 2). (ns) non-significant, * *p* < 0.05, ** *p* < 0.01 (two-tailed *t*-test). Data are mean values ± standard error (*n* = 4).

For the first time, the anti-inflammatory of EO of *C. amblyocarpa* has been evaluated via inhibition of the enzymes, lipoxygenase (LOX), cyclooxygenases (COX1, and COX2), as well as membrane stabilization. The results presented in Figure 6 revealed that the EO exhibited a significant anti-inflammatory action via the inhibition of LOX, COX1, and COX2 with respective IC_50_ values of 1.67, 12.77, 13.43 µg mL^−1^, whereas ibuprofen showed inhibition with IC_50_ values of 1.53, 10.26, and 12.71µg mL^−1^, respectively. Additionally, the EO significantly inhibited the lysis of the hypotonic solution of the RBCs at an IC_50_ value of 15.25 µg mL^−1^, compared with indomethacin which presented a result of 14.34 µg mL^−1^.

These data revealed that this EO has potent anti-inflammatory potentialities comparable with the two reference drugs, ibuprofen, and indomethacin, especially via the inhibition of lipoxygenase (LOX) and cyclooxygenase (COX1). This capability of EO for the inhibition of inflammations might be directly ascribed to the terpenoid contents as the main components (84.88%) [54]. Terpenoids represented major components of many of the documented plants with significant anti-inflammatory potentialities such as *Araucaria heterophylla* [8], *Ocimum basilicum* [55], and *Limnophila indica* [56].

Many studies have deduced that volatile sesquiterpene compounds have a potent anti-inflammation role in in vivo and in vitro models, especially caryophyllene and its oxide form [57]. Chavan et al. [58] described that the caryophyllene oxide, isolated from *Annona squamosa*, has significant in vivo anti-inflammatory activity. EO derived from *Cordia verbenacea* as well as its active constituent, caryophyllene, were demonstrated to exhibit strong anti-inflammatory activity was discussed with regard to their interfering with the production of TNF-*α* [59]. Moreover, Medeiros et al. [60] described that *trans*-caryophyllene showed potent anti-inflammatory in rats by significantly decreasing the migration of neutrophils as well as increasing the NF-κB-induced stimulation by lipopolysaccharides.

The EO of Sardinian *Santolina corsica* has been reported to contain a remarkable content of aromadendrene derivatives that exhibit a significant anti-inflammatory [61]. The enriched EO of Algerian *Myrtus communis* with myrtenyl acetate (38.7%) was shown to reduce the mice’s inflammation and paw edema at a concentration of 100 mg/kg [62]. In addition to these significant roles of the main components, the other compounds were expected to have an important contribution via synergetic effects [8]. Based upon all these reported data, it is very clear that the present data agree with the previously documented. Additionally, the present results could be attributed to the prevalence of sesquiterpenes, especially *trans*-caryophyllene and caryophyllene oxide.

## 3. Materials and Methods

### 3.1. Plant Materials

**Figure 7:**
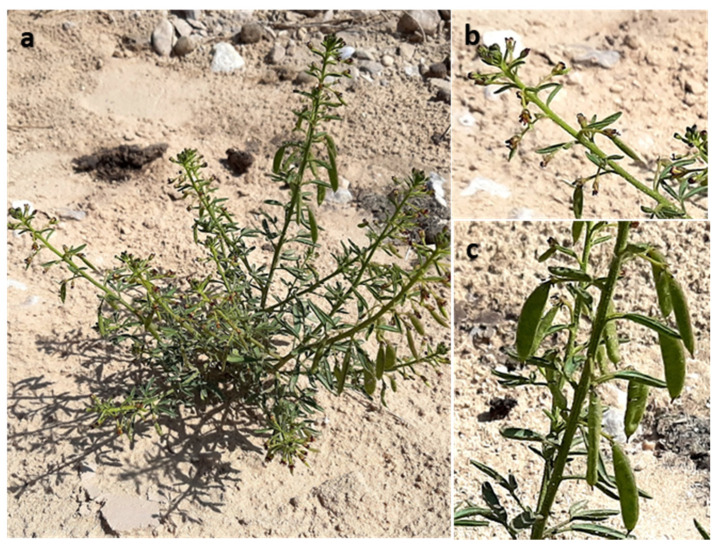
*Cleome amblyocarpa* Barratte & Murb. (**a**) Whole flowering plant, (**b**) enlarged flowering branch, and (**c**) enlarged fruiting branch. Photos were taken by Dr. Yasser El-Amier during spring of the year 2018 in Wadi Hajoul, eastern desert, Egypt.

Composite samples of the above-ground parts of the flowering *C. amblyocarpa* were collected in May 2018 from Wadi Hajoul, eastern desert, Egypt (29°57′51.2″ N 32°08′57.9″ E). The flowering plant was presented in Figure 7 The samples were collected from two populations in plastic bags, transferred to the laboratory, air-dried in a shaded place at room temperature for seven days, crushed into powder, and stored in paper bags until further analysis. The plant was identified according to [63,64]. A herbarium sheet (Mans.030301002) was prepared and deposited in the Herbarium of Botany Department, College of Science, Mansoura University, Egypt.

### 3.2. EO Extraction Analysis and Characterization

The EO chemical compositions of the two extracted EO samples were analyzed and identified separately by gas chromatography–mass spectrometry (GC-MS), as described in our previous publication [65].

### 3.3. Allelopathic Bioassay

The allelopathic activity of the extracted EO from the above-ground parts of *C. amblyocarpa* was evaluated in vitro against the weed, *Dactyloctenium aegyptium*. The ripe seeds of the weed were collected from a cultivated field near Gamasa city, northern Mediterranean coast, Egypt (31°26′19.3″ N 31°34′12.9″ E). The uniform seeds were surface-sterilized with sodium hypochlorite (0.3%), rinsed with distilled sterilized water, and dried under sterile conditions [34]. To test the allelopathic activity, serial concentrations (10–100 µg mL^−1^) of the EO were prepared using 1% polysorbate 80 (Sigma-Aldrich, Darmstadt, Germany) as an emulsifier. In a Petri plate, 20 sterilized seeds of *D. aegyptium* were arranged over wetted Whatman filter paper (Sigma-Aldrich, Darmstadt, Germany), either with each concentration or polysorbate 80 (as positive control). The plates were sealed with Parafilm^®^ tape (Sigma, St. Louis, MO, USA) and kept in a growth chamber adjusted at 25 °C.

The plates were checked every day, and after 7 days, the number of germinated seeds was counted, and the lengths (mm) of seedling roots and shoots were measured. The inhibition of the germination, root growth, and shoot growth of seedlings was calculated according to the following equation:

(1) $$Inhibition\;\left(\%\right)=100\times \left(\frac{Number/Length_{control}-(Number/Length_{treatment})}{Number/Length_{control}}\right)$$

Additionally, the IC_50_, which is the concentration of the EO required for 50% inhibition of seed germination or seedling growth, was calculated by linear regression of the inhibition values versus various EO concentrations using MS-EXCEL 2016.

### 3.4. Antioxidant Activity

The antioxidant activity of the EO from *C. amblyocarpa* was estimated based on its ability to reduce the stable radical, 2,2-diphenyl-1-picrylhydrazyl (DPPH). According to the method of Miguel [66], a reaction mixture of an equal volume of 0.3 mM of freshly prepared DPPH and serial concentrations (10–100 mg mL^−1^) of the EO or ascorbic acid as standard was prepared and well shaken. The mixture was incubated in dark conditions at room temperature (25 ± 2 °C) for 30 min. The absorbance was measured at 517 nm using a spectrophotometer (Spectronic 21D model). The scavenging activity was estimated according to the following equation:

(2) $$Scavengingactivity\left(\%\right)=100\times \left(1-\;\frac{Absorbance_{sample}}{Absorbance{\;}_{control}}\right)$$

The IC_50_, which is the concentration of the EO required to reduce the color of the DPPH by 50%, was calculated graphically by linear regression using MS-EXCEL 2016.

### 3.5. Anti-Inflammatory Activity Estimation

The anti-inflammatory activity of the EO from the above-ground parts of *C. amblyocarpa* was evaluated by assessing the in vitro membrane stabilizing, and inhibition of lipoxygenase (LOX, EC: 1.13.11.12) and cyclooxygenase (COX1 and COX 2, EC: 1.14.99.1) enzymes.

#### 3.5.1. Membrane Stabilization Inhibition Assay

The membrane-stabilizing activity of the samples was assessed using hypotonic solution-induced erythrocyte (RBCs) hemolysis [67]. For the preparation of erythrocyte suspension, whole blood was obtained with heparinized syringes from rats through cardiac puncture. The blood was washed three times with isotonic buffered solution (154 mM NaCl) in 10 mM sodium phosphate buffer (pH 7.4) and immediately centrifuged for 10 min at 3000× *g*. The test sample consisted of stock erythrocyte suspension (0.5 mL), 5 mL of hypotonic solution (50 mM NaCl), and the *C. amblyocarpa* EO (7.81–1000 µg mL^−1^ in ethanol) or indomethacin (as a standard drug). The control sample consisted of 0.5 mL of stock erythrocyte suspension and hypotonic-buffered saline solution alone. The mixtures were incubated for 10 min at room temperature (25 ± 2 °C) and centrifuged for 10 min at 3000× *g*. In 96-well plates, the absorbance of the supernatant was measured at 540 nm. The percentage inhibition of hemolysis or membrane stabilization were calculated according to the modified method described by Shinde et al. [67] as follows:

Inhibition % = 100 × [(A_control_ − A_treatment_) ÷ A_control_] (3)

where A_control_ is the absorbance control, and A_treatment_ is the absorbance treatment.

The IC_50_ value was defined as the concentration of the EO required to inhibit 50% of the RBC hemolysis under the assay conditions. It was calculated graphically by linear regression of the inhibition values of different concentrations using MS-EXCEL 2016.

#### 3.5.2. Lipoxygenase (LOX) Inhibition Assay

The activity of the *C. amblyocarpa* EO on the inhibition of the LOX enzyme (type I-B) was determined according to Granica et al. [68], with slight modifications. Briefly, in 96-well plates, 100 µL of soybean (*Glycine max*) LOX solution (1000 U/mL in borate buffer solution, pH 9) and 200 µL of borate buffer were mixed together with various concentrations of either EO (to a final concentration range of 0.98–125 µg mL^−1^) or ibuprofen as a reference drug. Samples were pre-incubated with 100 µL of linoleic acid (substrate) to initiate the reaction and then were incubated at 25 °C for 15 min. The absorbance increase was measured at 234 nm using a microplate reader (BioTek Instruments Inc., Winooski, VT, USA). The inhibition percentage and IC_50_ were calculated as previously mentioned in the membrane stabilization inhibition assay.

#### 3.5.3. Cyclooxygenase (COX 1 and COX 2) Inhibition Assay

The COX activity was monitored as a result of the oxidation reaction of N,N,N,N-tetramethyl-p-phenylenediamine (TMPD) with arachidonic acid according to the protocol of Petrovic and Murray [69], with slight modifications. The activity of the EO or ibuprofen as a reference drug at a concentration range of 0.98–125 µg mL^−1^ was determined by monitoring the absorbance of TMPD oxidation reaction with arachidonic acid at 611 nm using a microplate reader (BioTek Instruments Inc., Winooski, VT, USA). The inhibition percentage and IC_50_ were calculated as previously mentioned in the membrane stabilization inhibition assay.

### 3.6. Treatment of Data

The experiment of allelopathic bioassay was repeated three times with five replicas per each treatment. The data were subjected to one-way ANOVA followed by Tukey’s HSD at the probability level of 0.05 using CoStat software program, version 6.311 (http://www.cohort.com, CoHort, Monterey, CA, USA, 1 April 2017). Additionally, the antioxidant scavenging data were subjected to one-way ANOVA followed by Tukey’s HSD. The data of anti-inflammatory activity were compared using paired two-tailed *t*-tests using the XLSTAT 2018 program (https://www.xlstat.com/en/, Addinsoft Inc., New York, NY, USA, 15 January 2018). Chemometric analysis of the EO compositions of the studied Egyptian ecospecies in the present study and four other studied ecospecies collected from Saudi Arabia [28], Tunisia [37], Iran [27], and the United Arab Emirates [26] was performed via cluster analysis (agglomerative hierarchical clustering (AHC) and principal components analysis (PCA). We constructed a matrix of 65 compounds, from six samples of *C. amblyocarpa*, with a concentration >2%. The matrix was subjected to AHC and PCA using the XLSTAT 2018 program (https://www.xlstat.com/en/, Addinsoft Inc., New York, NY, USA, 15 January 2018).

## 4. Conclusions

For the first time, the analysis of the EO from the above-ground parts of the Egyptian ecospecies of *C. amblyocarpa* revealed the presence of 48 compounds, with a prevalence of sesquiterpenes. Caryophyllene oxide, hexahydrofarnesyl acetone, alloaromadendrene epoxide, myrtenyl acetate, isoshyobunone, shyobunol, and *trans*-caryophyllene have been identified as major compounds. The chemometric analysis of the presently studied ecospecies and other reported ecospecies revealed significant variation in the EO composition that could be ascribed to variation in the environmental and climatic conditions. EO showed substantial allelopathic inhibitory activity against the weed *D. aegyptium*, reflecting the potentiality of using this EO as an eco-friendly bioherbicide. Additionally, the EO showed significant antioxidant and anti-inflammatory activities. Further studies are recommended for evaluation (i) of anti-inflammatory effects of the *C. ambliocarpa* EO on an in vitro cell model (*ex*. RAW 264.7 cells); and (ii) the antioxidant, anti-inflammatory, and allelochemical activities along with the possible modes of action of the pure samples of the main EO compounds either singularly or in combination.

## Figures and Tables

**Table 1:** Chemical components of the essential oil of above-ground parts of *Cleome amblyocarpa*.

No.	RT ^a^	RC ^b^ %	Compound Name	MF ^c^	KI_Lit_^d^	KI_Exp_^e^	Identification ^f^
**Oxygenated monoterpenes**							
1	6.60	0.50	6-Camphenol	C_10_H_16_O	1113	1113	KI, MS
2	8.94	0.13	Eucalyptol	C_10_H_18_O	1031	1032	KI, MS
3	10.24	0.54	Camphor	C_10_H_16_O	1146	1165	KI, MS
4	11.00	0.45	Isopulegol	C_10_H_18_O	1149	1150	KI, MS
5	12.76	1.12	Borneol	C_10_H_18_O	1169	1169	KI, MS
6	11.99	0.25	2-ethyl-exo-Fenchol	C_10_H_18_O	1297	1299	KI, MS
7	26.43	5.73	Myrtenyl acetate	C_12_H_18_O_2_	1326	1324	KI, MS
**Sesquiterpenes hydrocarbons**							
8	16.34	0.13	Silphiperfol-5,7(14)-diene	C_15_H_22_	1360	1361	KI, MS
9	17.28	0.98	*α*-Copaene	C_15_H_24_	1376	1378	KI, MS
10	17.48	1.01	*β*-Maaliene	C_15_H_24_	1380	1381	KI, MS
11	17.70	0.47	Berkheyaradulene	C_15_H_24_	1388	1389	KI, MS
12	18.68	3.45	*trans*-Caryophyllene	C_15_H_24_	1408	1410	KI, MS
13	18.79	0.29	Widdrene	C_15_H_24_	1431	1430	KI, MS
14	19.22	0.46	*β*-Humulene	C_15_H_24_	1438	1436	KI, MS
15	19.85	0.49	*β*-Farnesene	C_15_H_24_	1442	1443	KI, MS
16	20.47	0.53	*γ*-Muurolene	C_15_H_24_	1479	1477	KI, MS
17	20.78	0.23	*ar*-Curcumene	C_15_H_22_	1480	1481	KI, MS
18	20.86	0.54	Valencene	C_15_H_24_	1496	1496	KI, MS
19	20.94	0.65	*α*-Selinene	C_15_H_24_	1498	1497	KI, MS
20	21.20	2.30	*α*-Muurolene	C_15_H_24_	1500	1500	KI, MS
21	21.78	1.17	*α*-Bulnesene	C_15_H_24_	1509	1510	KI, MS
22	22.00	1.02	*γ*-Cadinene	C_15_H_24_	1513	1514	KI, MS
23	23.55	0.42	*trans*-Calamenene	C_15_H_22_	1522	1520	KI, MS
24	32.80	1.01	*α*-Guaiene	C_15_H_24_	1600	1601	KI, MS
**Oxygenated sesquiterpenes**							
25	20.21	0.13	*β*-Cubebene	C_15_H_24_O	1388	1389	KI, MS
26	21.53	1.53	*trans*-*α*-Bisabolene epoxide	C_15_H_24_O	1506	1508	KI, MS
27	21.67	1.54	*trans*-Nerolidol	C_15_H_24_O	1531	1533	KI, MS
28	23.17	4.52	Isoshyobunone	C_15_H_24_O	1571	1570	KI, MS
29	23.48	0.66	Spathulenol	C_15_H_24_O	1577	1579	KI, MS
30	23.78	36.01	Caryophyllene oxide	C_15_H_24_O	1582	1584	KI, MS
31	24.22	0.86	Humulene oxide	C_15_H_24_O	1608	1609	KI, MS
32	24.45	0.83	Junenol	C_15_H_26_O	1619	1621	KI, MS
33	24.53	0.56	Citronellyl valerate	C_15_H_28_O_2_	1625	1626	KI, MS
34	24.63	1.18	Nerolidol-epoxyacetate	C_17_H_28_O_4_	1638	1637	KI, MS
35	24.88	0.38	tau-Cadinol	C_15_H_26_O	1640	1639	KI, MS
36	25.34	6.17	Alloaromadendrene epoxide	C_15_H_24_O	1641	1643	KI, MS
37	25.57	0.71	*α*-Eudesmol	C_15_H_26_O	1653	1655	KI, MS
38	25.80	0.32	Aromadendrene oxide-(2)	C_15_H_24_O	1678	1680	KI, MS
39	25.99	4.19	Shyobunol	C_15_H_24_O	1688	1691	KI, MS
40	26.79	0.49	*β*-Santalol	C_15_H_24_O	1738	1735	KI, MS
41	29.32	0.54	Xanthorrhizol	C_15_H_22_O	1753	1752	KI, MS
**Diterpenes hydrocarbons**							
42	33.47	0.22	Geranyl-α-terpinene	C_20_H_32_	1874	1872	KI, MS
**Oxygenated diterpenes**							
43	37.49	0.17	Phytol	C_20_H_40_O	1942	1944	KI, MS
**Carotenoid derived compounds**							
44	14.56	1.39	Theaspirane A	C_13_H_22_O	1305	1307	KI, MS
45	14.87	1.64	Dihydroedulan II	C_13_H_22_O	1318	1316	KI, MS
**Apocarotenoid derived compounds**							
46	30.87	7.92	Hexahydrofarnesyl acetone	C_18_H_36_O	1835	1837	KI, MS
**Other compounds**							
47	13.87	1.16	*p*-Isopropyl-benzaldehyde	C_10_H_12_O	1239	1240	KI, MS
48	38.11	0.18	9,12-Octadecadienoic acid	C_18_H_32_O_2_	2085	2085	KI, MS
**Total**		97.17					

^**a**^ RT: Retention time. ^**b**^ RC: Relative concentration. **^c^** MF: Molecular formula. **^d^** KI_Lit_: Kovats retention index according to Adams (2017) on a DB−5 column in reference to *n*-alkanes. **^e^** KI_Exp_: Experimental calculated Kovats retention index. **^f^** EO constituent identification was constructed via compound mass spectra (MS) and Kovats retention indices (KI) with those of Wiley spectral library collection and NIST library databases.

## Data Availability

Not applicable.

## Sample Availability

Samples of the compounds are not available from the authors.
